# Use of freeze-dried umbilical cord amniotic membrane as a corneal patch graft

**DOI:** 10.1016/j.ajoc.2025.102477

**Published:** 2025-11-08

**Authors:** Anna Bistour, David Mrejen, Louis Breyton, Jean-Louis Bourges

**Affiliations:** Department of Ophthalmology, Centre Hospitalier Universitaire Cochin, Assistance Publique-Hopitaux de Paris, Université Paris Cité, Paris, France

## Abstract

**Background:**

The use of amniotic membranes for corneal perforation management has been extensively documented, demonstrating significant potential in ocular surface reconstruction. However, their application in emergency settings remains underexplored. This report presents a novel use of freeze-dried umbilical cord amniotic membrane as a corneal patch graft, highlighting its practical benefits in urgent clinical scenarios.

**Case report:**

A 64-year-old male presented to the emergency department with a painful, red eye, having a history of penetrating keratoplasty. Slit-lamp examination revealed a severe corneal abscess with a 3 mm-wide paracentral perforation. Emergency surgery was performed using a freeze-dried umbilical cord amniotic membrane (SclerFix, Amtrix®, TBF) with a “mushroom plug and patch” technique, combined with a fresh amniotic membrane overlay. Postoperative treatment included oral antibiotics, topical antifungal agents, and antibiotic eye drops. The fresh amniotic membrane dissolved within days, while the freeze-dried umbilical cord membrane facilitated rapid re-epithelialization, effectively sealing the perforation. At three months postoperatively, the cornea remained intact without recurrence or complications, and the patient reported complete pain resolution.

**Conclusions:**

This case demonstrates the successful use of freeze-dried umbilical cord amniotic membrane as a corneal patch graft, avoiding emergency keratoplasty and preserving ocular integrity. This technique represents a promising alternative in situations where corneal grafts are not readily available. Further studies are warranted to validate its efficacy.

## 1 Background

Amniotic membrane grafts have been widely used in ophthalmology for ocular surface reconstruction.^1^^,^^2^ Initially, only cryopreserved amniotic membranes were available, but the introduction of freeze-dried variants has facilitated broader clinical use due to improved accessibility and shelf-life.^3^

Traditionally, only the deepest layer of the amniotic membrane, rich in collagen, fibronectin, and laminin, was used, measuring 30–50 μm in thickness. Subsequently, thicker amniotic membranes incorporating the spongy layer, enriched in proteoglycans, were introduced, reaching approximately 150 μm.^4^ More recently, lyophilized patches of umbilical cord amnion overlaid with Wharton's jelly have been developed, yielding a thickness of 1–2 mm (SclerFix, Amtrix®, TBF).^5^

While these patches have been studied for scleral thinning,^6^ their application in emergency corneal perforation repair has not yet been explored. Corneal perforation management varies based on etiology and severity, incorporating techniques such as cyanoacrylate glue, corneal grafting, conjunctival flaps, and amniotic membrane transplantation.^7^^,^^8^ Although cyanoacrylate glue is highly inflammatory and full-thickness keratoplasty presents challenges in availability and rejection risk, amniotic membrane grafts have demonstrated success in sealing corneal perforations.^9^ Various techniques, including multilayer and inlay-onlay sandwich methods, have been described,^10^^,^^11^ but none have yet examined the use of freeze-dried umbilical cord amniotic membrane in this context.

This case report describes the use of freeze-dried umbilical cord amniotic membrane as a corneal patch in the emergency management of an infectious corneal perforation.

## 2 Case report

A 64-year-old male presented to our emergency department with a left painful, red eye. His medical history included a prior penetrating keratoplasty performed over 20 years ago for advanced keratoconus, with good graft survival and stability over the intervening years. The patient had no significant systemic comorbidities.

Ophthalmologic examination revealed best-corrected visual acuity of light perception in the affected eye. Slit-lamp biomicroscopy demonstrated a corneal abscess with a 3 mm-wide paracentral perforation extending toward the graft–host interface, a positive Seidel test, absence of the anterior chamber, and corectopia directed towards the corneal defect. Although no clear elements were present in the patient's history such as trauma with vegetative matter, the clinical presentation with a dense infiltrate, feathery margins, and deep stromal involvement strongly suggested a fungal etiology.

A diagnosis of perforated corneal abscess was established, and the patient was immediately hospitalized for emergency surgery under general anesthesia.

**Fig. 1:**
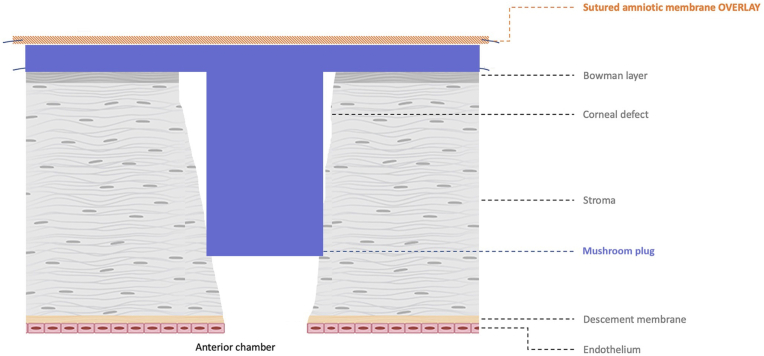
Schematic representation of the mushroom plug and patch technique.

Following sampling of the abscess for microbiological testing, a “mushroom plug and patch” technique was employed using a freeze-dried umbilical cord amniotic membrane (SclerFix, Amtrix®, TBF) (Fig. 1). The “mushroom” structure was created by inserting the base of the membrane into the perforation while allowing the cap to extend over the defect. The graft was secured with 10-0 nylon sutures.

To enhance ocular surface protection, a fresh amniotic membrane overlay was sutured to the conjunctiva using 8-0 Vicryl. The patient was hospitalized for 48 hours and received intravenous levofloxacin and both antifungal and antibiotic eyedrops. Upon discharge, treatment included a four-week regimen of antifungal (amphotericin B/voriconazole) and antibiotic (tobramycin) eye drops, along with oral levofloxacin for 10 days.

**Fig. 2:**
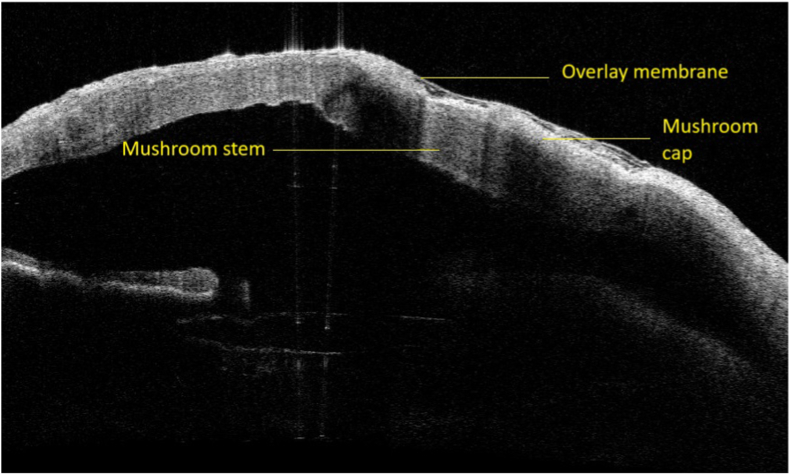
Anterior segment optical coherence tomography one week after surgery visualizing the different layers and confirming the position of the mushroom plug.

**Fig. 3:**
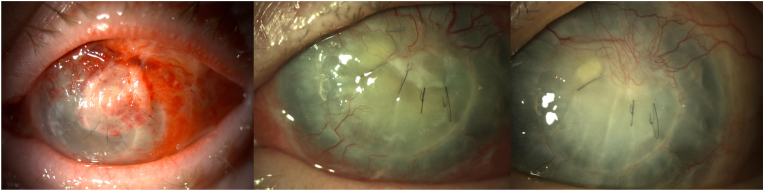
Evolution of the mushroom plug one week, one month and three months after surgery from left to right.

Postoperative follow-up at one week demonstrated stable graft integration with no recurrence of perforation (Fig. 2). The fresh amniotic membrane overlay began dissolving, revealing rapid re-epithelialization and early conjunctivalization at the perforation site. Exposed sutures were removed during follow-up. By three months postoperatively, the patient was pain-free, visual acuity remained at light perception, and slit-lamp examination confirmed a healed cornea with a well-formed anterior chamber, resolution of the previously observed corectopia and no Seidel sign (Fig. 3).

## 3 Discussion

This case illustrates an innovative application of freeze-dried umbilical cord amniotic membrane for the management of an infectious corneal perforation. The described technique has not been previously reported for this indication. While amniotic membrane transplantation is well-documented for ocular surface reconstruction, its use as an emergency corneal patch graft has remained unexplored.

This case involved a corneal perforation likely caused by a virulent infectious agent, facilitated by ocular surface instability, the immune-compromised and structural fragility of the long-standing graft. Despite negative microbiological results, the clinical features of the corneal abscess including its dense stromal infiltrate, feathery margins, and rapid progression, were strongly suggestive of a fungal etiology. Infectious corneal melting can rapidly lead to severe ocular morbidity, including globe rupture if not promptly treated.

The primary goal in this case was to control the infection while preserving ocular integrity. Freeze-dried umbilical cord amniotic membrane, with its anti-inflammatory, anti-fibrotic, and anti-angiogenic properties, was selected as the primary grafting material.^3^^,^^5^ Unlike cryopreserved membranes, which require specialized storage, freeze-dried membranes offer extended shelf-life and room-temperature storage, making them highly accessible for emergency cases.

The increased thickness and biomechanical properties of umbilical cord amniotic membrane facilitated better defect coverage and integration compared to standard amniotic membranes. Thanks to its intrinsic properties, this technique results in less inflammation than cyanoacrylate glue and supports more effective healing. The “mushroom plug and patch” technique allowed stable graft adherence and rapid tissue regeneration, preventing further corneal melting and preserving globe integrity.

While umbilical cord amniotic membrane is known to possess anti-fibrotic properties,^5^ it may appear paradoxical in the context of corneal wound healing, which relies in part on stromal remodeling and fibrosis for structural restoration. The rapid re-epithelialization and apparent stromal integration observed in this case suggest that additional cellular and molecular processes are at play. The exact mechanisms by which umbilical cord amniotic membrane supports corneal healing, particularly in the setting of full-thickness defects, remain incompletely understood. It is possible that umbilical cord amniotic membrane modulates the activity of resident keratocytes or promotes a controlled remodeling response that balances regenerative healing. Further experimental studies are needed to elucidate the physiopathological pathways involved.

## 4 Conclusion

This case highlights the successful application of freeze-dried umbilical cord amniotic membrane for the treatment of corneal perforation, offering a practical alternative to emergency keratoplasty. The technique demonstrates a promising approach for corneal repair in resource-limited or urgent settings. Further studies are needed to fully assess its long-term efficacy and clinical outcomes.

## CRediT authorship contribution statement

**Anna Bistour:** Writing – original draft. **David Mrejen:** Investigation. **Louis Breyton:** Writing – original draft. **Jean-Louis Bourges:** Writing – review & editing.

## Patient consent

Written consent to publish this case has not been obtained. This report does not contain any personal identifying information.

## Authorship

All authors attest that they meet the current ICMJE criteria for Authorship.

## Funding

No funding or grant support

## Declaration of competing interest

The authors declare that they have no known competing financial interests or personal relationships that could have appeared to influence the work reported in this paper.
